# Transradial intra-aortic catheter looping in the angioplasty of severe intracranial symptomatic arteriosclerotic diseases

**DOI:** 10.3389/fneur.2023.1226306

**Published:** 2023-10-13

**Authors:** Gang-Qin Xu, Jin-Chao Xia, Dong-Yang Cai, Bo-Wen Yang, Tong-Yuan Zhao, Jiang-Yu Xue, Zi-Liang Wang, Tian-Xiao Li, Bu-Lang Gao

**Affiliations:** ^1^Cerebrovascular Disease Hospital of Henan Provincial People's Hospital, Zhengzhou, Henan, China; ^2^Endovascular Intervention Center, Zhengzhou University People's Hospital, Zhengzhou, Henan, China; ^3^Henan Provincial International Joint Laboratory of Cerebrovascular Diseases, Zhengzhou, Henan, China

**Keywords:** transradial access, intra-aortic guiding catheter looping, atherosclerotic stenosis, occlusion, large intracranial arteries, angioplasty

## Abstract

**Purpose:**

This study aims to investigate the effect and feasibility of intra-aortic catheter looping via transradial access in angioplasty for symptomatic intracranial severe (>70%) atherosclerotic stenosis or occlusion of large arteries (SISOLAs).

**Materials and methods:**

Patients with SISOLAs who underwent transradial endovascular angioplasty using the catheter looping technique in the ascending aorta were retrospectively enrolled. The clinical data and treatment outcomes were analyzed.

**Results:**

Fifteen patients aged 48–71 years were enrolled in this study. Left vertebrobasilar artery occlusion was present in 1 (6.7%) patient, severe left middle cerebral artery stenosis in 7 (46.7%) patients, severe left internal carotid artery (ICA) stenosis of the ophthalmic segment in 4 (26.7%) patients, severe left ICA stenosis of the cavernous segment in 2 (13.3%) patients, and severe right middle cerebral artery stenosis in 2 (13.3%) patients. The arterial stenosis ranged from 70 to 92% (mean 86%) before stenting. The looping of a guiding catheter in the ascending aorta via transradial access for angioplasty was successful in all patients (100%). The vertebral artery intracranial segment occlusion was successfully recanalized, while severe stenosis in the remaining 14 patients was successfully eliminated. After endovascular recanalization, the residual stenosis was reduced by 12–26% (median 18%). No puncture-related complications or surgical-related neurological complications occurred in these patients. In the follow-up angiography conducted on 10 (66.7%) patients after 6–25 months, no in-stent restenosis was detected.

**Conclusion:**

Intra-aortic guiding catheter looping via transradial access for endovascular angioplasty of SISOLAs is technically safe, feasible, and effective, especially when the transfemoral artery approach is difficult or impossible to undertake.

## Introduction

Due to increased comfort, shorter hospital stay, and fewer bleeding complications at the puncture location, transradial artery access is increasingly being applied currently in the endovascular diagnosis and treatment of coronary heart diseases, as compared to transfemoral access (1–6). For cerebral angiography and endovascular treatment of cerebrovascular diseases, the use of transradial access has also been on the rise (7–11). Successful endovascular treatment through transradial access depends on navigating a guiding catheter to the proper location with good support for endovascular management; however, not all patients have good arterial anatomy for a successful and smooth endovascular operation. In patients with a bovine aortic arch, where the left and right common carotid arteries originate from the same trunk, the arterial angle formed between the common carotid artery and the innominate or subclavian artery is very acute and is not good for the smooth navigation of the guiding catheter to the right location for an endovascular operation (12, 13). In patients with a lower origin of the vertebral artery, those with a need for superselecting the contralateral vertebral artery, or those with a type III aortic arch, in which all the arteries on the arch originate below a straight line, which leads to the formation of an acute angle between the arterial origin and the aortic arch (12–15), huge difficulties are frequently encountered when attempting to navigate the guiding catheter to the proper location or maintain its stability for performing the endovascular treatment using conventional access approaches. Thus, intra-aortic catheter looping with the guiding catheter forming a big loop inside the ascending aorta via radial artery puncture and access (transradial access) may be of great help for endovascular treatment. This technique has been applied successfully for cerebral angiography and endovascular treatment of cerebrovascular diseases (13, 16–21). Nonetheless, these studies primarily used the intra-aortic catheter looping technique for the treatment of carotid artery stenting (16–19), cerebral angiography (20), aortic arch lesions (21), and cerebral aneurysms (13). No studies have been performed using the intra-aortic catheter looping technique for the treatment of symptomatic intracranial severe atherosclerotic stenosis (>70%) or occlusion of large arteries (SISOLAs). It was hypothesized that this technique could also be used via transradial access for the angioplasty of SISOLAs. Thus, this study was conducted to test this hypothesis.

## Materials and methods

### Subjects

This retrospective one-center study was approved by the ethics committee of our hospital, and all patients provided written informed consent for participation. Between January 2016 and April 2023, patients with SISOLAs were included. The inclusion criteria were patients with SISOLAs who underwent angioplasty via the transradial intra-aortic catheter looping technique and due to difficulties encountered through transfemoral access for angioplasty. The exclusion criteria were patients with SISOLAs treated through transfemoral access for intracranial angioplasty.

### Technical skills

All patients underwent a modified Allen test (22) for the assessment of the collateral circulation function before endovascular treatment, and the test results were negative.

**Figure 1:**
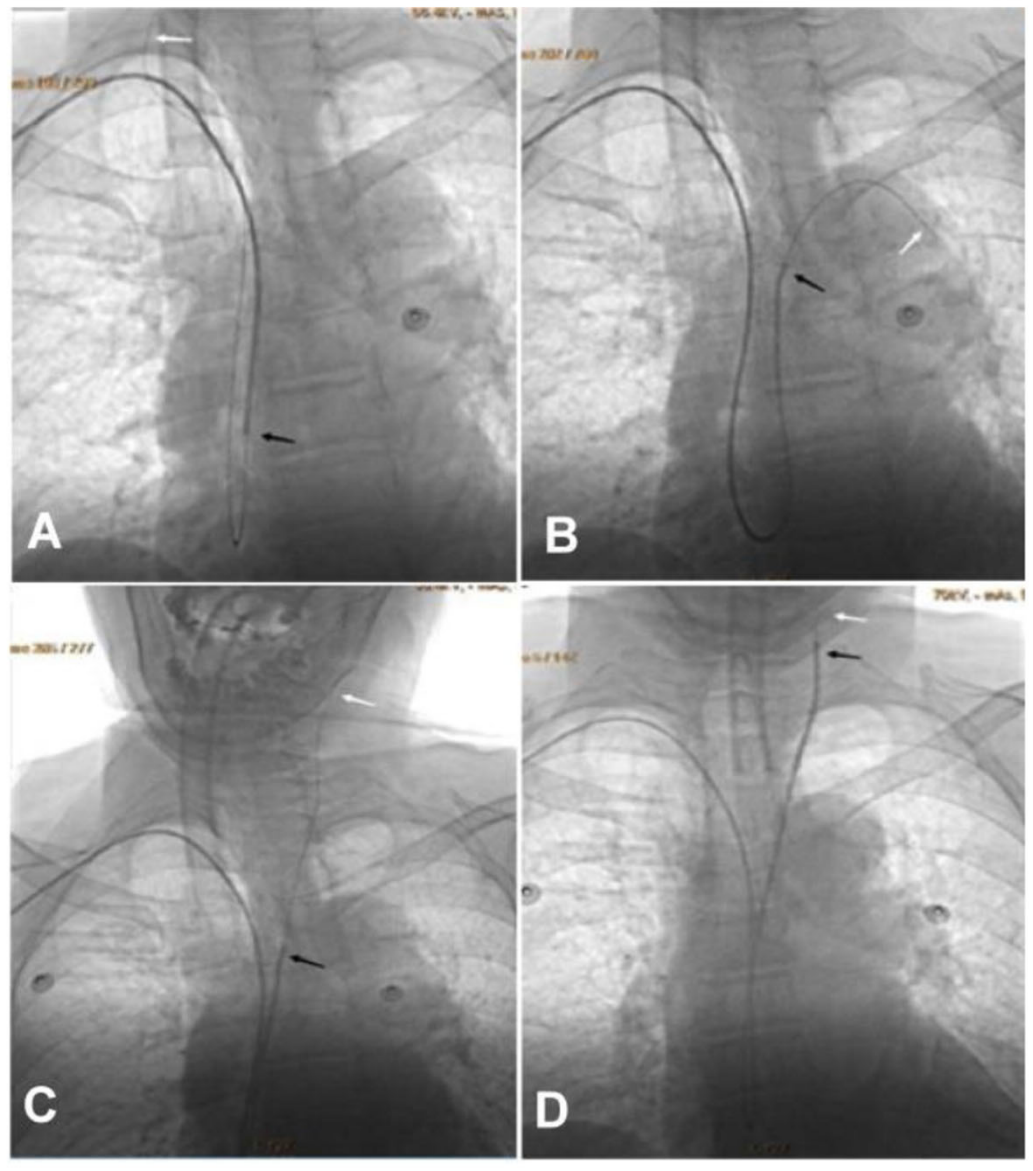
The intra-aortic catheter looping technique through transradial access is shown. **(A)** A loach guide wire was sent into the aorta for looping. **(B)** The guiding catheter followed the guide wire and formed a loop within the aorta. **(C)** The loach guide wire was used to superselect the target artery. **(D)** The guiding catheter was navigated into the target artery with the help of the loach guide wire. Black arrow, the tip of the guiding catheter; white arrow, the tip of the loach guide wire.

**Figure 2:**
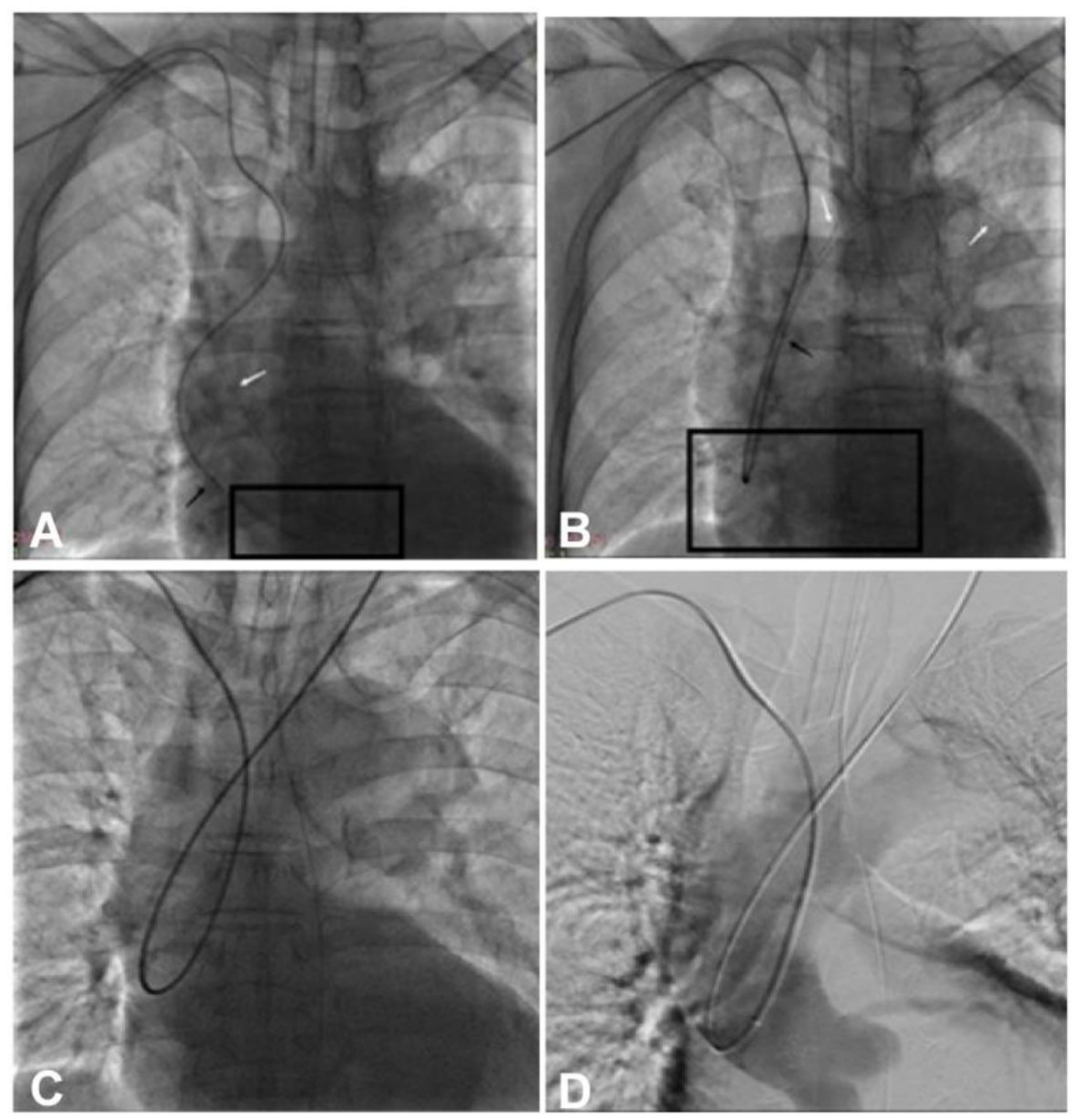
A guiding catheter and a guide wire were looped in the ascending aorta. **(A)** The guide wire was looped at the aortic valve. The box indicates the loop of the guide wire. **(B, C)** After the loop was formed, it was lifted upward away from the aortic valve. **(D)** Angiography revealed that the looped guide wire and guiding catheter were supported by the aortic wall rather than by the aortic valve. Black arrow, the tip of the guiding catheter; white arrow, the tip of the loach guide wire.

**Figure 3:**
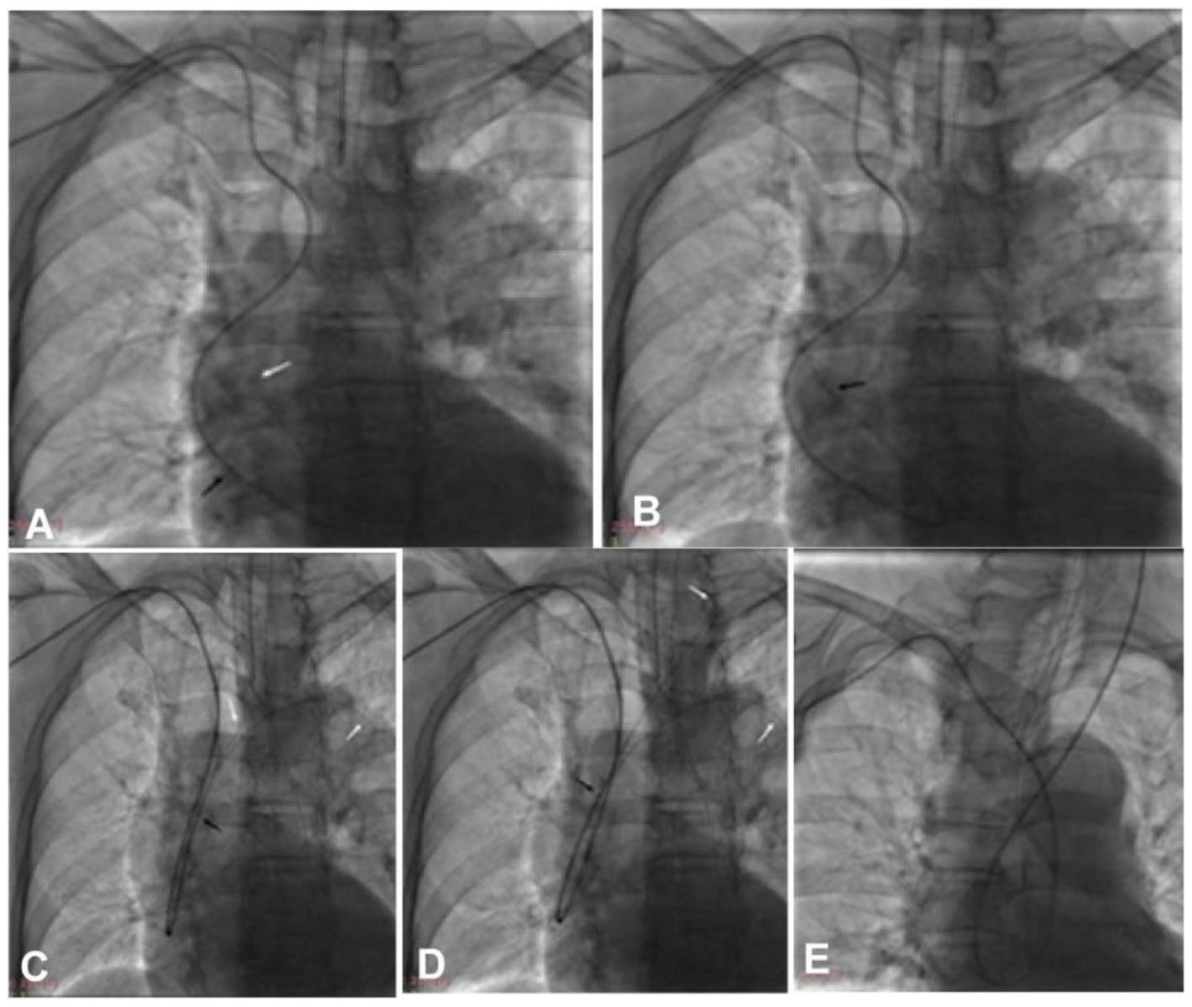
The double-loach technique was used for catheter looping within the aorta via transradial access. **(A)** A loach guide wire was sent into the aortic valve to form a loop. **(B)** The guiding catheter was navigated via the loach guide wire into the aorta to form a loop. **(C)** The tip of one loach guide wire was sent into the descending aortic lumen, while the tip of another loach guide wire was used to superselect the target artery. **(D)** The loach guide wire was sent into the target artery. **(E)** The guiding catheter was sent into the target artery via the loach guide wire. Black arrow, the tip of the guiding catheter; white arrow, the tip of the loach guide wire.

The endovascular procedure was conducted under general anesthesia, and the right radial artery was punctured for the insertion of a 6F arterial sheath. A total of 5,000 units of heparin and 200 μg of nitroglycerin were administered through the sheath. A SIM2 catheter (Cordis, Miami Lakes, FL, USA) was preferentially selected for cerebral angiography to evaluate the relevant lesion (stenosis or occlusion) and the collateral circulation. Then, a 0.035-in 150 cm loach guide wire (Vietnam, TERUMO, Japan) was used to guide a 0.70-inch Navien 115/125 cm long (Medtronic, USA) or a 6F ENVOY (Cordis Chihuahua, Mexico, USA) guiding catheter through the subclavian artery into the ascending aorta for looping. The loach guide wire was first looped at the aortic valve before carefully navigating the guiding catheter through the looped guide wire to form a loop within the ascending aorta (Figures 1, 2). Then, the tip of the looped guiding catheter was used to superselect the target artery (Figures 1, 2). If the target artery was difficult to superselect, a double-loach guide wire technique was used (Figure 3). The tip of one loach guide wire was sent into the descending aorta and adjusted to reach the origin of the target artery, while another loach guide wire was sent into the origin of the target artery before navigating the guiding catheter through the guide wire into the target artery (Figure 3). The guiding catheter tip was located proximal to the lesion before withdrawing the loach guide wire. If an intermediate catheter was used, a V18 guide wire (COSTARICA, Boston Scientific, MA, USA) was inserted into the target artery before withdrawing the loach guide wire. The V18 guide wire enhanced the Navien catheter support and prevented it from folding at the looping site. After establishing the treatment pathway, balloon dilation, and stent implantation were performed for intracranial vascular stenosis/occlusion lesions. After confirming good vascular reconstruction through angiography, the surgery was terminated by withdrawing the endovascular treatment system.

### Statistical analysis

In this study, statistical analysis was conducted with the SPSS software version 20.0 (IBM, Chicago, IL, USA). Measurement data were presented as mean and standard deviation if they were normally distributed or as median and interquartile range if they were non-normally distributed. Categorical data were presented as frequencies and percentages and tested with the Chi-squared test. The statistically significant p-level was set at <0.05.

## Results

Fifteen patients were enrolled, including 9 (60%) male and 6 (40%) female patients, with an age range of 48–71 (mean 58) years (Table 1). Eight (53.3%) patients changed their choice from the transfemoral artery approach to the transradial access, while 7 patients chose the transradial artery approach because of difficulty in treatment through transfemoral access on preoperative evaluation. In all 15 patients, it was either difficult to guide a guiding catheter for superselection to the proper location by the conventional transradial access approach or the guiding catheter was unstable and could easily herniate into the aorta after reaching the superselection position during angioplasty in routine transradial endovascular treatment.

**Table 1:** Demography and clinical data.

**Variables**	**Data**
Age (y)	48–71 (mean 58)
M/F	9/6
Hypertension (*n*, %)	12 (80%)
Diabetes mellitus (*n*, %)	6 (40%)
Hyperlipidemia (*n*, %)	10 (66.7%)
Smoking (*n*, %)	9 (60%)
Abdominal aortic/femoral/iliac artery occlusion or severe stenosis (*n*, %)	4 (26.7%)
Severe tortuosity of the abdominal aorta (*n*, %)	4 (26.7%)
Type III aortic arch (*n*, %)	7 (46.7%)
Ankylosing spondylitis (*n*, %)	1 (6.7%)

Comorbidities included hypertension in 12 (80%) patients, diabetes mellitus in 6 (40%) patients, hyperlipidemia in 10 (66.7%) patients, and smoking in 9 (60%) patients (Table 1). Negative conditions predisposing the difficulty or impossibility of endovascular treatment included concurrent occlusion or severe stenosis (70%) of the abdominal aorta, femoral or iliac artery in 4 (26.7%) patients, severe tortuosity of the abdominal aorta in 4 (26.7%) patients, type III aortic arch in 7 (46.7%) patients, and ankylosing spondylitis in 1 (6.7%) patient.

Among the 15 patients (Table 2), left vertebrobasilar artery occlusion was present in 1 (6.7%) patient, severe left middle cerebral artery stenosis in 7 (46.7%) patients, severe left internal carotid artery (ICA) stenosis of the ophthalmic segment in 4 (26.7%) patients, severe left ICA stenosis of the cavernous segment in 2 (13.3%) patients, and severe right middle cerebral artery stenosis in 2 (13.3%) patients. In one patient (6.7%), severe stenosis was present in the left ICA cavernous segment and left-middle cerebral artery.

**Table 2:** Treatment of intracranial stenosis or occlusion and outcomes.

**Variables**		**Data**
Intracranial lesions (*n*, %)	Left vertebrobasilar artery occlusion	1 (6.7%)
	Left middle cerebral artery stenosis	7 (46.7%)
	Left ICA ophthalmic segment stenosis	4 (26.7%)
	Left ICA cavernous segment stenosis	2 (13.3%)
	Right-middle cerebral artery stenosis	2 (13.3%)
Transradial access (*n*, %)	Right radial artery	15 (100%)
Treatment approach (*n*, %)	Balloon dilation + stent implantation	14 (93.3%)
	Balloon dilation alone (n, %)	1 (6.7%)
Complications	Puncture-related complications (hematoma and radial artery occlusion) (*n*, %)	0
	Approach-related neurological complications (cerebral infarction, hemorrhage, vasospasm, and dissection) (*n*, %)	0

ICA, internal carotid artery.

**Figure 4:**
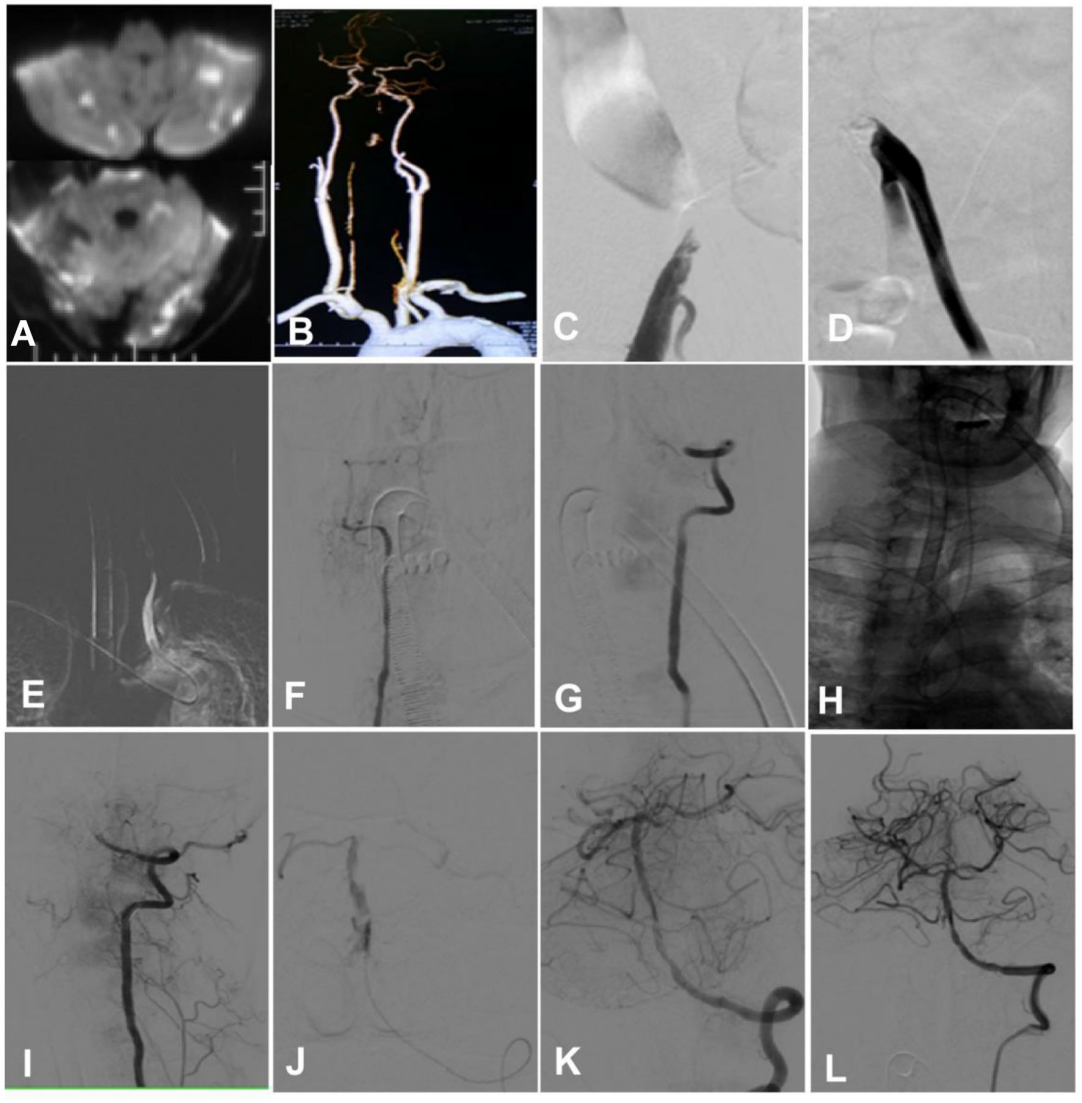
A man in his early 50s with hypertension for 20 years and diabetes mellitus for 20 years was admitted because of tetraplegia for 12 h and dizziness and diplopia for 2 days. **(A)** A magnetic resonance imaging scan showed multiple fresh infarcts in the bilateral cerebellum, the occipital lobe, and the brain stem. **(B)** Computed tomographic angiography revealed bilateral vertebral artery intracranial segment occlusion, with no display of the basilar artery. **(C)** Angiography through the right femoral artery demonstrated occlusion of the right iliac artery. **(D)** Angiography via the left femoral artery indicated occlusion of the left iliac artery. **(E–G)** Cerebral angiography through the right radial artery angiography showed occlusion of the bilateral vertebral artery intracranial segment. **(H)** After accessing through the right radial artery, a guiding catheter was sent to superselect the left vertebral artery after looping in the aorta. **(I)** The tip of the guiding catheter was navigated to the V3 segment of the left vertebral artery. **(J)** With the help of a Synchro-14 micro-guide wire (Stryker, Salt Lake City, Utah, USA), an Echelon-10 microcatheter (Medtronic, Heerlen, Netherlands, USA) was navigated through the occluded segment for angiography, and it showed good patency of the distal arteries. **(K, L)** A Gateway 2.0 × 15 mm balloon catheter (Stryker Fremont, CA, USA) was used to dilate the occluded segment before deploying an Enterprise 4.5 × 22 mm stent (Cordis, Chaska, MN, USA) at the segment. Angiography revealed good blood flow in the vertebral and basilar arteries.

The endovascular treatment was performed all through the right radial artery, and a guiding catheter was successfully looped in the ascending aorta for angioplasty in all patients (100%). The vertebral artery intracranial segment occlusion was successfully recanalized (Figure 4), while the severe stenoses in the other 14 patients were all successfully eliminated. The arterial stenosis ranged from 70 to 92% (mean 86%) before stenting, and the residual stenosis was 12–26% (median 18%) after endovascular angioplasty. During the endovascular operation, the guiding catheter remained stable. The heart rate and blood pressure of the patients were stable, and no puncture-related complications or surgical-related neurological complications occurred. Follow-up angiography was performed in 10 (66.7%) patients 6–25 months (median 13) after stenting. No in-stent restenosis was detected.

## Discussion

In this study, which investigated the effect and feasibility of intra-aortic catheter looping via transradial access for angioplasty for the treatment of SISOLAs, it was found that intra-aortic guiding catheter looping via transradial access for endovascular angioplasty of SISOLAs is technically safe, feasible, and effective, especially when endovascular treatment is difficult or impossible to perform through the transfemoral artery approach.

For cerebrovascular diagnosis and treatment via transradial access, the guiding catheter should first go downward to pass through the subclavian/innominate artery and then enter the aorta before turning backward and upward to enter the target vessel. Most cerebral angiography via the transradial artery approach can be performed only by using a reverse-curved catheter to select the artery's origin through “rotation and lifting,” without the need for further superselection. The operation is relatively simple. Nonetheless, when transradial access is applied for endovascular embolization of intracranial aneurysms, there are high requirements for the guiding catheter: (1) the target vessels must be superselected, and the catheter tip should be sent as close to the lesion as possible and (2) the guiding catheter should have a stable support point on the vascular wall to provide sufficient support for an endovascular operation. For patients with a blunt angle between the subclavian/innominate artery and the common carotid artery, such as in the case of “superselecting the left carotid artery in the bovine arch through the right radial artery,” the guiding catheter can be easily sent to the proper location with good support (12). However, when the angle between the subclavian/innominate artery and the common carotid artery is acute, it is relatively difficult to send the catheter in place, and a harder guiding catheter is required to change the local vascular morphology in order to form a stable support point on the vascular wall (23). For patients with sharp angles, patients with a large distance between the innominate artery and the left common carotid artery, or those with severe calcification of the aorta and arterial branches above the arch, it is usually difficult to navigate a harder guiding catheter to the proper location with good support without causing injury to the arteries (23, 24).

The use of the intra-aortic catheter looping technique reportedly easily overcomes the aforementioned anatomical difficulties in the endovascular treatment of cerebrovascular diseases (13, 16–20). After the guiding catheter forms a big loop inside the ascending aorta, the top of the catheter loop is supported by the aortic top wall, thus providing strong support to the guiding catheter for further endovascular operations (13, 16–20). After looping, the tip of the looped guiding catheter is consistent with the direction of blood flow, and thus, it is relatively easy to superselect branches of blood vessels above the aortic arch. In studies on the literature, the endovascular exchange technique has been employed for looping the guiding catheter inside the aorta: a small-diameter catheter was first looped inside the aorta to superselect the target artery. Then, a hard exchange guide wire was used to guide a larger-diameter guiding catheter or a long sheath along the guide wire to form a loop inside the aorta before entering the target artery (16–20). If the support force of a single guide wire is insufficient, the double guide wires technique or the balloon anchoring technique is required (16, 19, 21, 25).

In our study, a 6F guiding catheter or a softer Navien catheter was used instead of a large-diameter guiding catheter or a long sheath for successful catheter looping inside the aorta under the guidance of a loach guide wire alone. This operation is simple and convenient, and the target artery can be easily superselected without using more complex exchange techniques or causing frictional injury to the arterial wall. For cases with difficulty superselecting the target blood vessels, two loach guide wires can be used for superselection, thus greatly improving the efficiency of blood vessel selection. For patients with tortuous common carotid and internal carotid arteries, the use of an intermediate catheter is preferred for easy positioning. However, since the intermediate catheter itself is soft and has weak axial support, a V18 guide wire can be placed inside the guiding catheter (guide wire retention technique) to enhance the catheter support and avoid catheter breakage after the intermediate catheter is in place. The inner lumen of the 6F intermediate catheter is large enough to ensure that the placement of a V18 guide wire does not affect subsequent operations.

There are some conditions and precautions for the intra-aortic catheter looping technique. The guiding catheter must be of sufficient length for catheter looping inside the ascending aorta. Thus, a 100-cm guiding catheter can reach the V3 segment for posterior circulation lesions if the patient's height is ≤ 175 cm. If the patient's height is >175 cm or the guiding catheter needs to be sent to a high intracranial location, a 115-cm intermediate catheter needs to be used. For anterior circulation lesions, a 115-cm intermediate catheter is frequently required. If the patient's height is >180 cm or the catheter needs to be sent to a high intracranial location, a 125-cm intermediate catheter is needed. Severe tortuosity of the subclavian artery and the innominate artery may increase the path length to a certain extent but not significantly. Especially when the guiding catheter is in place, the tortuosity of the subclavian and innominate arteries is straightened, and the guiding catheter basically becomes the shortest path for treatment. Thus, the tortuosity of the upper limb and innominate arteries has little effect on the catheter's length. The endovascular path's length mainly depends on the length of the upper limb and that of the ascending aorta. These two factors are directly proportional to the height of the patient, making it easy and convenient to judge the length of the catheter based on the patient's height. Nonetheless, this method is prohibited for patients with aortic valve insufficiency, valve excrescence, or ascending aorta diseases because of the fear of aggravating the conditions. The use of a harder catheter for intra-aortic looping may have catastrophic consequences, such as aortic injury.

Some limitations existed in this study, including the retrospective one-center study design, lack of randomization and control, a small patient cohort, and the enrolment of only Chinese patients, which might all affect the generalization of the outcome. Future prospective, randomized, controlled, multicenter studies will have to be conducted with multiple races and ethnicities involved to obtain better outcomes.

In conclusion, intra-aortic guiding catheter looping via transradial access for endovascular angioplasty of SISOLAs is technically safe, feasible, and effective, especially when the transfemoral artery approach is difficult or impossible to undertake.

## Data availability statement

The original contributions presented in the study are included in the article/supplementary material, further inquiries can be directed to the corresponding author.

## Ethics statement

The studies involving humans were approved by Ethics Committee of Henan Provincial People's Hospital. The studies were conducted in accordance with the local legislation and institutional requirements. The participants provided their written informed consent to participate in this study.

## Author contributions

Study design: G-QX and B-LG. Data collection: G-QX, J-CX, D-YC, B-WY, T-YZ, J-YX, and Z-LW. Writing of the original version: G-QX. Revision and editing: B-LG. All authors contributed to the article and approved the submitted version.

## References

[1] Alnasser SM Bagai A Jolly SS Cantor WJ Dehghani P Rao SV. Transradial approach for coronary angiography and intervention in the elderly: A meta-analysis of 777,841 patients. Int J Cardiol. (2017) 228:45–51. 10.1016/j.ijcard.2016.11.207 27863361

[2] Jolly SS Yusuf S Cairns J Niemela K Xavier D Widimsky P. Radial versus femoral access for coronary angiography and intervention in patients with acute coronary syndromes (rival): A randomised, parallel group, multicentre trial. Lancet. (2011) 377:1409–20. 10.1016/S0140-6736(11)60404-2 21470671

[3] Rao SV Ou FS Wang TY Roe MT Brindis R Rumsfeld JS. Trends in the prevalence and outcomes of radial and femoral approaches to percutaneous coronary intervention: A report from the national cardiovascular data registry. JACC Cardiovasc Interv. (2008) 1:379–86. 10.1016/j.jcin.2008.05.007 19463333

[4] Cao G Cai HX Cao J. Advancement in coronary angiography or percutaneous coronary intervention using the distal transradial artery access in acute coronary syndrome and complex coronary artery disease. Anatol J Cardiol. (2022) 26:163–71. 10.5152/AnatolJCardiol.2021.933 35346902 PMC9366409

[5] Bice F Eyuboglu M Ozmen ZC Acikel B Yilmaz M Karayakali M. The relationship between radial artery spasm and adropin levels in patients undergoing transradial coronary angiography. J Cardiovasc Thorac Res. (2022) 14:90–4. 10.34172/jcvtr.2022.15 35935383 PMC9339735

[6] Stephan T Gierl MT Felbel D Rattka M Rottbauer W Gonska B. Vascular access-site choice and outcomes in patients with previous coronary artery bypass surgery undergoing coronary catheterization in a high-volume transradial center. J Invasive Cardiol. (2022) 34:E237–48. 35235530 10.25270/jic/21.00022

[7] Virk HUH Ullah W Ahmed M Chattarjee S Witzke CF Banka S. Transradial versus transfemoral artery catheterization: A comparative meta-analysis on cerebrovascular accidents. Expert Rev Cardiovasc Ther. (2021) 19:103–5. 10.1080/14779072.2021.1860752 33290666

[8] Hendrix P Melamed I Weiner GM Goren O Griessenauer CJ Schirmer CM. Transradial versus transfemoral intraoperative cerebral angiography for open cerebrovascular surgery: Effectiveness, safety, and learning curve. Oper Neurosurg. (2023) 24:476–82. 10.1227/ons.0000000000000567 36701679

[9] Shaban S Rastogi A Phuyal S Huasen B Haridas A Zelenak K. The association of transradial access and transfemoral access with procedural outcomes in acute ischemic stroke patients receiving endovascular thrombectomy: A meta-analysis. Clin Neurol Neurosurg. (2022) 215:107209. 10.1016/j.clineuro.2022.107209 35290788

[10] Kuroiwa M Hanaoka Y Koyama JI Yamazaki D Kubota Y Kitamura S. Transradial mechanical thrombectomy using a radial-specific neurointerventional guiding sheath for anterior circulation large-vessel occlusions: Preliminary experience and literature review. World Neurosurg. (2023) 171:e581–9. 10.1016/j.wneu.2022.12.060 36529427

[11] Alkhars H Haq W Al-Tayeb A Sigounas D. Feasibility and safety of transradial aneurysm embolization: A systematic review and meta-analysis. World Neurosurg. (2022) 165:e110–27. 10.1016/j.wneu.2022.05.112 35654332

[12] Gao BL Xu GQ Wang ZL Li TX Wang YF Liang XD. Transradial stenting for carotid stenosis in patients with bovine type and type iii aortic arch: experience in 28 patients. World Neurosurg. (2018) 111:e661–7. 10.1016/j.wneu.2017.12.138 29294393

[13] Xu GQ Ba YY Cai DY Yang BW Zhao TY Xue JY. Transradial access with intra-aortic catheter looping for the treatment of intracranial aneurysms. Front Neurol. (2023) 14:1128960. 10.3389/fneur.2023.1128960 37181573 PMC10174240

[14] Chen SH Snelling BM Shah SS Sur S Brunet MC Starke RM. Transradial approach for flow diversion treatment of cerebral aneurysms: a multicenter study. J Neurointerv Surg. (2019) 11:796–800. 10.1136/neurintsurg-2018-014620 30670622

[15] Chivot C Bouzerar R Yzet T. Transitioning to transradial access for cerebral aneurysm embolization. Am J Neuroradiol (AJNR). (2019) 40:1947–53. 10.3174/ajnr.A6234 31582386 PMC6975100

[16] Fang HY Chung SY Sun CK Youssef AA Bhasin A Tsai TH. Transradial and transbrachial arterial approach for simultaneous carotid angiographic examination and stenting using catheter looping and retrograde engagement technique. Ann Vasc Surg. (2010) 24:670–9. 10.1016/j.avsg.2009.12.001 20363587

[17] Lee WC Fang HY Chen HC Hsueh SK Fang CY Chen CJ. Comparison of a sheathless transradial access with looping technique and transbrachial access for carotid artery stenting. J Endovasc Ther. (2016) 23:516–20. 10.1177/1526602816640291 27004495

[18] Ruzsa Z Nemes B Pinter L Berta B Toth K Teleki B. A randomised comparison of transradial and transfemoral approach for carotid artery stenting: Radcar (radial access for carotid artery stenting) study. EuroIntervention. (2014) 10:381–91. 10.4244/EIJV10I3A64 25042266

[19] Wu CJ Cheng CI Hung WC Fang CY Yang CH Chen CJ. Feasibility and safety of transbrachial approach for patients with severe carotid artery stenosis undergoing stenting. Catheter Cardiovasc Interv. (2006) 67:967–71. 10.1002/ccd.20738 16649240

[20] Wu CJ Hung WC Chen SM Yang CH Chen CJ Cheng CI. Feasibility and safety of transradial artery approach for selective cerebral angiography. Catheter Cardiovasc Interv. (2005) 66:21–6. 10.1002/ccd.20396 16082678

[21] Zhang J Liu X Tian M Chen H Wang J Ji M. Endovascular aortic repairs combined with looping-chimney technique for repairing aortic arch lesions and reconstructing left common carotid artery. J Thorac Dis. (2020) 12:2270–9. 10.21037/jtd.2020.04.31 32642132 PMC7330391

[22] Agostoni P Biondi-Zoccai GG de Benedictis ML Rigattieri S Turri M Anselmi M. Radial versus femoral approach for percutaneous coronary diagnostic and interventional procedures; systematic overview and meta-analysis of randomized trials. J Am Coll Cardiol. (2004) 44:349–56. 10.1016/j.jacc.2004.04.034 15261930

[23] Khan NR Peterson J Dornbos Iii D Nguyen V Goyal N Torabi R. Predicting the degree of difficulty of the trans-radial approach in cerebral angiography. J Neurointerv Surg. (2021) 13:552–8. 10.1136/neurintsurg-2020-016448 32792364

[24] Choi SW Kim S Kim H Kim SR Park IS. Anatomical predictors of difficult left internal carotid artery navigation in transradial access for neurointervention. J Neurosurg. (2022) 139:157–164. 10.1101/2021.10.07.21264684 36334297

[25] Liang XD Wang ZL Li TX He YK Bai WX Wang YY. Safety and efficacy of a new prophylactic tirofiban protocol without oral intraoperative antiplatelet therapy for endovascular treatment of ruptured intracranial aneurysms. J Neurointerv Surg. (2016) 8:1148–53. 10.1136/neurintsurg-2015-012055 26614492

